# Comparing the diagnostic accuracy of Afirma GSC to ThyroSeq V3 in cytologically indeterminate thyroid nodules

**DOI:** 10.1530/ETJ-25-0296

**Published:** 2025-12-09

**Authors:** Natasha Dowell, Shayma Begum, Jameel Muzaffar, Kristien Boelaert, Hannah Nieto

**Affiliations:** ^1^ College of Medicine and Health, University of Birmingham, Birmingham, UK; ^2^ Department of Ear, Nose and Throat Surgery, University Hospitals Birmingham NHS Foundation Trust, Birmingham, UK; ^3^ Department of Applied Health, College of Medicine and Health, University of Birmingham, Birmingham, UK; ^4^ Department of Metabolism and Systems Science, College of Medicine and Health, University of Birmingham, Birmingham, UK

**Keywords:** thyroid, thyroid nodule, thyroid cancer

## Abstract

**Objective:**

To compare the diagnostic test accuracy of Afirma GSC and ThyroSeq v3 in cytologically indeterminate thyroid nodules.

**Methods:**

PubMed, Embase, Cochrane Library, Medline, and the Web of Science were searched from the date of inception to May 9, 2025. Two independent reviewers screened articles for eligibility. Studies assessing Afirma gene sequencing classifier (GSC) or ThyroSeq v3 for indeterminate thyroid nodules were included. The Preferred Reporting Items for Systematic Reviews and Meta-Analysis reporting guidelines were followed. Statistical analysis was performed using R.

**Results:**

A total of 26 studies met the eligibility criteria. For surgically confirmed Afirma GSC results, the sensitivity, specificity, and negative predictive value (NPV) were 94, 42, and 96%, respectively. For unoperated negative cases, the values were 96, 86, and 99%. For ThyroSeq v3, the sensitivity, specificity, and NPV for surgically confirmed cases were 96, 40, and 93%, respectively, and for unoperated negative cases, they were 97, 83, and 99%.

**Conclusion:**

Both molecular tests demonstrate high NPV but low specificity; neither is clearly superior. Future research should prioritise randomised controlled trials, long-term follow-up of unoperated nodules, and direct comparisons of molecular tests.

## Introduction

Thyroid nodules are estimated to be present in 6–67% of the population, and while most are benign, 10–15% are found to be malignant (1, 2). Fine-needle aspiration cytology (FNAC) is the standard investigation for classifying thyroid nodules as benign or malignant, providing management guidance (3). A limitation of FNAC is that an estimated 25% of patients will receive a cytologically indeterminate result, meaning it is uncertain if the nodule is benign or malignant (4). Indeterminate thyroid nodules include Bethesda III (atypia of undetermined significance or follicular lesion of undetermined significance) and IV (follicular neoplasm or suspicious for follicular neoplasm) (5). In most indeterminate cases, the patient will undergo diagnostic hemithyroidectomy so the nodule can be examined histologically for a final diagnosis. The malignancy rate of operated indeterminate nodules is reported at 33.9%, meaning many benign nodules are operated on (6). This poses a significant burden on healthcare systems through unnecessary surgeries, which can also negatively impact patients’ quality of life.

The American Thyroid Association recommends molecular testing to guide the management of cytologically indeterminate thyroid nodules (7). These tests aim to reduce unnecessary diagnostic surgeries and optimise patient outcomes. There are several commercially available molecular tests. Afirma Genomic Sequencing Classifier (GSC), (developed by Veracyte Inc, USA) analyses mRNA expression to determine malignant potential, and represents a development from the previous generation, Afirma gene expression classifier (GEC) (8). ThyroSeq v3 genomic classifier uses next-generation sequencing to assess 112 gene alterations associated with thyroid carcinoma (9). Both tests report samples as benign or suspicious.

Previous meta-analyses have primarily focused on individual molecular tests or earlier test generations (10, 11, 12, 13, 14). Two previous reviews compared the diagnostic accuracy between ThyroSeq v3, Afirma GSC, and microRNA panels. Both reviews had a strong methodology; however, they were both limited by their sample size (15, 16).

A previous review assessed the real-world performance of Afirma GSC by assuming unoperated molecular test negative cases are true negatives. This was because most patients in these studies did not have surgery (and final histology), with a negative GSC result, but instead had follow-up on ultrasound for 6–12 months assessing nodule growth (11). No previous review has analysed the performance of ThyroSeq v3 in this way. Our review assesses real-world performance of Afirma GSC and ThyroSeq v3 by analysing diagnostic performance in both surgically confirmed nodules and unoperated molecular test negative (assumed true negative) nodules. This approach provides a more comprehensive understanding of the diagnostic performance of these molecular tests and addresses gaps in the existing literature.

## Materials and methods

This systematic review and meta-analysis was performed according to the Preferred Reporting Items for Systematic Reviews and Meta-Analysis (PRISMA) guidelines (17). Before the literature screening, this review was prospectively registered through the International Prospective Register of Systematic Reviews (PROSPERO) (Registration number: CRD42024578531).

### Literature search and data collection

A description of the participants, interventions, comparisons, outcomes, timing, and study design is provided in Table 1. The following databases were searched from date of inception to May 9, 2025: PubMed, Embase, Cochrane Central Register of Controlled Trials (CENTRAL), OVID MEDLINE, and the Web of Science. The search terms were designed with the support of a research librarian (Table 2). Additional texts were identified through reference searching of the included studies. Articles were screened for eligibility by two independent reviewers (ND, SB). Any discrepancies were resolved through discussion with a third independent reviewer (HN).

**Table 1:** Population, interventions, comparisons, outcomes, timing, study design (PICOTS).

PICOTS	Description
Population	All patients (no age limitation) with cytologically indeterminate thyroid nodules where the sample was adequate but not possible to differentiate between benign and malignant disease
Interventions	Afirma GSC or ThyroSeq version 3 with histopathologic diagnosis
Comparisons	Each other
Outcomes	Diagnostic accuracy of the molecular test
Timing	Any time point
Study design	A systematic review and meta-analysis

**Table 2:** PubMed search terms.

PubMed	(‘Indeterminate’[title/abstract] OR ‘Thy3’[title/abstract] OR ‘Thy4’[title/abstract] OR ‘bethesda category 3’[title/abstract] OR ‘bethesda category 4’[title/abstract] OR ‘bethesda category iii’[title/abstract] OR ‘bethesda category iv’[title/abstract]) AND (‘ThyroSeq’[title/abstract] OR ‘Afirma’[title/abstract] OR ‘Affirma’[title/abstract] OR ‘GSC’[title/abstract] OR ‘molecular diagnostic’[title/abstract] OR ‘genetic testing’[title/abstract] OR ‘molecular marker’[title/abstract] OR ‘genom*’[title/abstract] OR ‘ThyGenX’[title/abstract] OR ‘ThyGeNEXT’[title/abstract] OR ‘ThyraMIR’[title/abstract] OR ‘Rosetta’[title/abstract] OR ‘ThyroSpec’[title/abstract] OR ‘miRInform’[title/abstract] OR ‘AmpliSeq’[title/abstract] OR ‘mir-THYpe’[title/abstract] OR ‘gene sequencing classifier’[title/abstract] OR ‘next generation sequencing’[title/abstract] OR ‘cytopathology’[title/abstract]) AND (‘thyroid gland’[title/abstract] OR ‘thyroid nodule’[title/abstract] OR ‘thyroid neoplasms’[title/abstract] OR ‘thyroid cancer’[title/abstract] OR ‘thyroid carcinoma’[title/abstract] OR ‘thyroid microcarcinoma’[title/abstract] OR ‘thyroid tum*’[title/abstract] OR ‘thyroid adenoma’[title/abstract] OR ‘thyroid adenocarcinoma’[title/abstract] OR ‘thyroid nod*’[title/abstract] OR ‘thyroid lump’[title/abstract] OR ‘thyroid swelling’[title/abstract] OR ‘follicular’[title/abstract] OR ‘papillary’[title/abstract] OR ‘malignant’[title/abstract]) AND (‘biopsy, fine needle’[MeSH terms] OR (‘biopsy’[all fields] AND ‘fine needle’[all fields]) OR ‘fine-needle biopsy’[all fields] OR (‘fine’[all fields] AND ‘needle’[all fields] AND ‘aspiration’[all fields]) OR ‘fine needle aspiration’[all fields] OR (‘biopsy, fine needle’[MeSH terms] OR (‘biopsy’[all fields] AND ‘fine needle’[all fields]) OR ‘fine-needle biopsy’[all fields] OR (‘fine’[all fields] AND ‘needle’[all fields] AND ‘biopsy’[all fields]) OR ‘fine needle biopsy’[all fields]))

### Eligibility criteria

Inclusion criteria:

– All patients (no age limitation) with cytologically indeterminate thyroid nodules (Bethesda III or IV).
– Articles must evaluate either ThyroSeq V3 or Afirma GSC as an index test.
– The article must contain true positive (TP), false positive (FP), false negative (FN), and true negative (TN) results to calculate the sensitivity, specificity, positive predictive value (PPV), and negative predictive value (NPV).
– All patients must have histopathological confirmation.

Exclusion criteria:

– Duplicates, review articles, comments, conference abstracts, case reports, and unpublished articles.
– Studies with missing data regarding TP, FP, TN, or FN.
– Older-generation molecular tests or next generation sequencing platforms, for example, but not limited to: Afirma GEC, ThyroSeq v2, ThyraMIR, ThyGeNEXT, or multiplatform tests.
– Studies not published in the English language.

Non-invasive follicular thyroid neoplasm with papillary-like nuclear features (NIFTP) was categorised as malignant by most included studies, and therefore this review classified them as such, despite these lesions not being considered malignant (16). There was no limitation on year of publication. The reference lists of all included articles and any relevant systematic reviews were screened by the reviewers.

Older-generation molecular tests, such as Afirma GEC and ThyroSeq v2, were excluded from this review, as our aim was to evaluate the most current versions to provide a true update to the existing literature. These earlier models have already been extensively discussed in prior reviews. Similarly, newly emerging next-generation sequencing and multiplatform tests were also excluded due to limited data availability and lack of direct comparability.

### Data extraction

Two independent reviewers (ND, SB) extracted the relevant data using a predefined Excel (Microsoft, USA) table. Any discrepancies were resolved by discussion with a third reviewer (HN). The following data were extracted from each article:

– Study characteristics: first author, publication year, country.
– Patient characteristics: participant number, patient selection method, mean age, male:female ratio.
– Outcome data: TPs, FPs, TNs, and FNs, sensitivity, specificity, PPV, NPV.

### Study quality

Two independent reviewers (ND and SB) assessed the studies’ methodological quality using the Downs and Black checklist (18). Studies were categorised as excellent, good, fair, or poor quality.

### Statistical analysis

For each article, the TP, FP, TN, and FN were used to calculate the sensitivity, specificity, PPV, and NPV. Sensitivity and specificity, with their 95% confidence intervals, were pooled using a bivariate random-effects model. All statistical analysis was performed with R version 4.4.3 using the packages ‘Meta’, ‘Mada’, ‘ggplot2’, ‘pROC’, and ‘Metafor’ (R Project for Statistical Computing, Vienna, Austria) (19, 20, 21, 22, 23, 24). Study heterogeneity was quantified using the *I*^2^ statistic. *P* values ≤0.05 were considered statistically significant. Studies with nodules with a positive molecular test result that did not undergo surgery were excluded from the calculations. Studies with nodules with a negative molecular test that did not undergo surgery were recorded separately for comparison.

## Results

### Study characteristics

**Figure 1:**
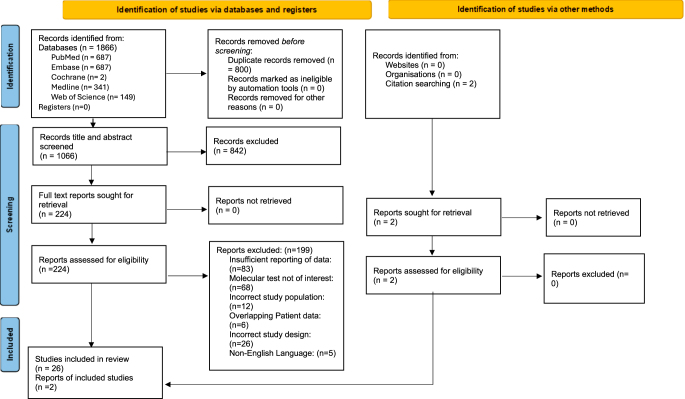
Preferred reporting items for systematic reviews and meta-analyses (PRISMA) flowchart.

Initial searches yielded 1,866 studies (Fig. 1). Eight hundred duplicates were removed, and 1,066 were screened at the title and abstract stage, leaving 224 for full-text review. Following screening, 26 studies identified through database searches and two identified through citation searching were included in the review. Eight studies were multi-centre, and 18 were based at a single centre. Patient and study characteristics are provided in eTables 2 and 3 (see section on Supplementary materials given at the end of the article). Publication years ranged from 2015 to 2025. The mean age of patients who had Afirma GSC and ThyroSeq v3 testing was 53.6 and 54.7 years, respectively. A total of 1,127 and 1,162 surgically confirmed nodules were assessed using ThyroSeq v3 and Afirma GSC, respectively. Twenty-one studies were based in the USA, one study was based in the USA and Singapore, one study from the Netherlands, one from Singapore, and one from Canada. One study was a randomised controlled trial, 7 were prospective, and 18 contained historical data.

### Quality and publication bias

The quality assessment scores using the Downs and Black checklist are provided in eTable 4. The scores ranged from 12 to 22 out of a total of 28. Five studies were categorised as good quality, 17 as fair quality, and four were of poor quality. The main reason behind the range in quality assessment was the lack of randomisation across studies.

### Results regarding the diagnostic performance of the molecular panels

#### Afirma GSC: operated nodules with histological diagnosis

**Figure 2:**
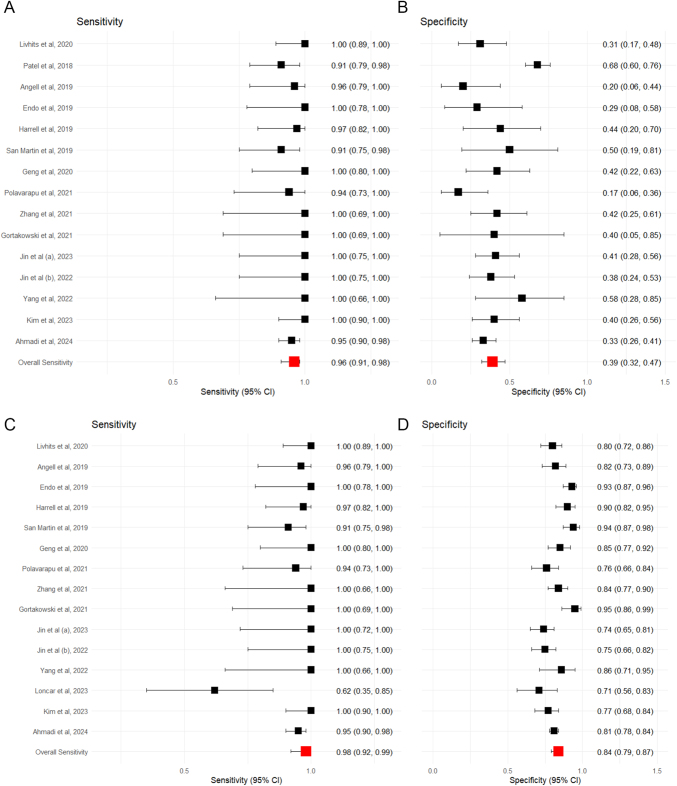
Forest plot showing the sensitivity (A) and specificity (B) for Afirma GSC, where only surgically confirmed cases are included, and sensitivity (C) and specificity (D) assuming all unoperated test-negative cases are true negatives. Squares represent the point estimate for an individual study. The line represents the 95% confidence interval. The red square represents the overall pooled effect.

Sixteen studies, including 1,162 surgically confirmed nodules, evaluated Afirma GSC (5, 25, 26, 27, 28, 29, 30, 31, 32, 33, 34, 35, 36, 37, 38, 39). Surgically confirmed cases had a range of sensitivities from 0.91 to 1.00, eTable 5. Specificity ranged from 0.17 to 0.68. Within this surgically confirmed Afirma GSC subgroup, the pooled estimates of sensitivity and specificity were 0.94 (95% CI: 0.91–0.97) and 0.42 (95% CI: 0.27–0.59), respectively, as shown in Fig. 2. The negative predictive value (NPV) was 0.96 (95% CI: 0.89–0.98). Pooled summary data is provided in Table 3.

**Table 3:** Pooled summary data. The 95% CI values are provided in parentheses.

	SC-Afirma GSC	Afirma GSC*	SC-ThyroSeq v3	ThyroSeq v3*
Sensitivity	0.96 (0.91–0.98)	0.98 (0.92–0.99)	0.96 (0.94–0.97)	0.97 (0.92–0.99)
Specificity	0.39 (0.32–0.47)	0.84 (0.79–0.87)	0.40 (0.33–0.47)	0.83 (0.79–0.87)
NPV	0.96 (0.89–0.99)	1.00 (0.98–1.00)	0.93 (0.85–0.96)	0.99 (0.98–1.00)

* Assuming all test-negative unoperated cases are true negatives.

NPV, negative predicted value; SC, surgically confirmed.

#### Afirma GSC: non-operated nodules with assumed negative diagnosis

**Figure 3:**
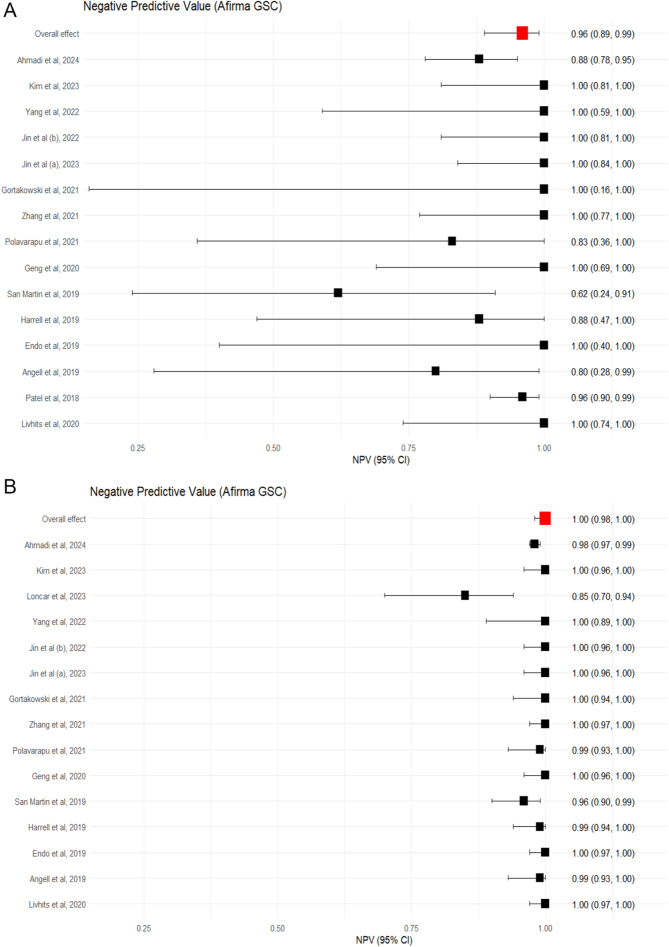
Forest plot showing the NPV for Afirma GSC as a molecular test, where only surgically confirmed cases are included (A), and assuming all unoperated test-negative cases are true negatives (B). Squares represent the point estimate for an individual study. The line represents the 95% confidence interval. The red square represents the overall pooled effect.

Sixteen studies reported test-negative cases that did not undergo surgical intervention, eTable 6, (5, 26, 27, 28, 29, 30, 31, 32, 33, 34, 35, 36, 37, 38, 39). Assuming all test-negative cases that were not operated on were true-negatives, the sensitivity ranged from 0.63 to 1.00, and specificity ranged from 0.71 to 0.95. The pooled estimates of sensitivity and specificity were 0.96 (95% CI: 0.92–0.98) and 0.86 (95% CI: 0.81–0.90), respectively, Fig. 2. The negative predictive value was 0.99 (95% CI: 0.98–1.00), which was expected when assuming all unoperated cases are true negatives, Fig. 3.

Loncar *et al.* did not specify the number of surgically confirmed true negatives in their GSC cohort; they assumed that a negative ultrasound at 6 months meant the nodule was a true negative, and therefore its results were not included in the surgically confirmed cases (39).

#### ThyroSeq V3 next-generation sequencing: operated nodules with histological diagnosis

**Figure 4:**
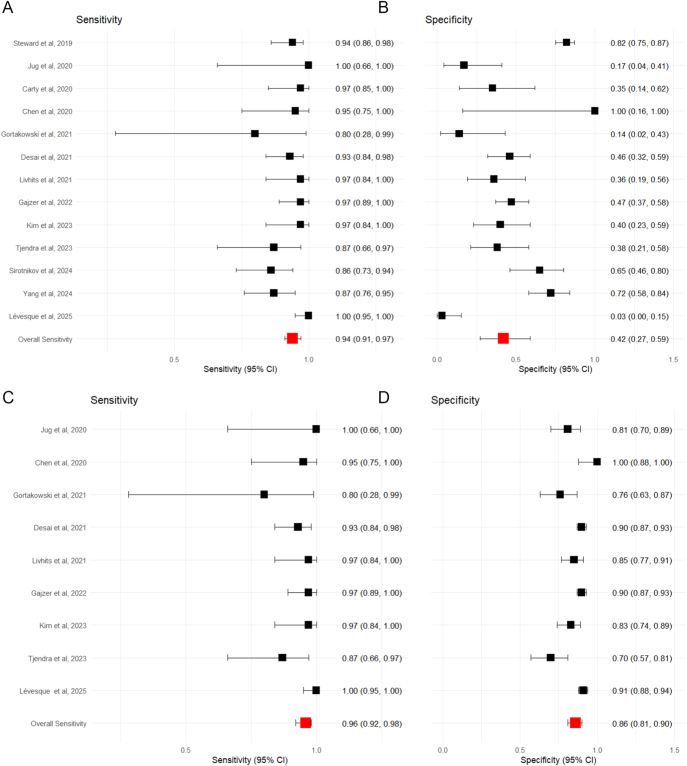
Forest plot showing the sensitivity (A) and specificity (B) for ThyroSeq v3, where only surgically confirmed cases are included, and sensitivity (C) and specificity (D) assuming all unoperated test-negative cases are true negatives. Squares represent the point estimate for an individual study. The line represents the 95% confidence interval. The red square represents the overall pooled effect.

**Figure 5:**
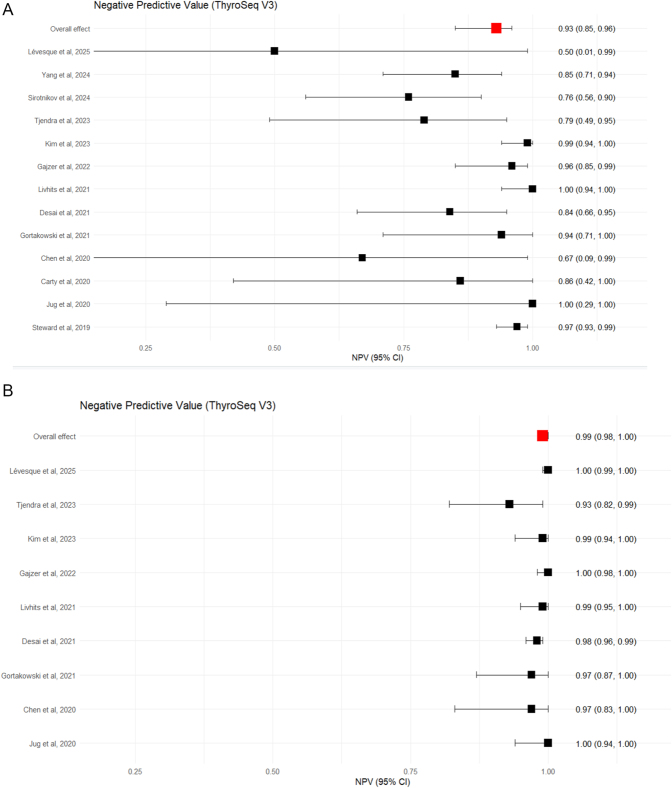
Forest plot showing the NPV for ThyroSeq v3, where only surgically confirmed cases are included (A), and assuming all unoperated test-negative cases are true negatives (B). Squares represent the point estimate for an individual study. The line represents the 95% confidence interval. The red square represents the overall pooled effect.

Thirteen studies, comprising 1,127 surgically confirmed nodules, assessed the performance of ThyroSeq V3 (5, 9, 26, 34, 40, 41, 42, 43, 44, 45, 46, 47, 48). Cases with confirmed surgical follow-up demonstrated a range of sensitivities from 0.80 to 1.00, eTable 7. Specificity ranged from 0.03 to 1.00. Within this surgically confirmed subgroup, the pooled estimates of sensitivity and specificity were 0.96 (95% CI: 0.94–0.97) and 0.40 (95% CI: 0.33–0.47) respectively, Fig. 4. The negative predictive value was calculated as 0.93 (95% CI: 0.85–0.96), Fig. 5.

#### ThyroSeq V3 next generation sequencing: non-operated nodules with assumed negative diagnosis

Nine studies reported test-negative cases that were not operated on (5, 26, 34, 40, 42, 43, 44, 45, 46, 48). Assuming all unoperated test-negative cases were true negatives, the sensitivities ranged from 0.8 to 1.00, eTable 8. Specificity ranged from 0.76 to 1.00. The pooled estimates of sensitivity and specificity were 0.97 (95% CI: 0.92–0.99) and 0.83 (95% CI: 0.79–0.87), respectively, Fig. 4. The negative predictive value was 0.99 (95% CI: 0.98–1.00), which was as expected when assuming all unoperated cases are true negatives, Fig. 5.

#### Comparing Afirma GSC and GEC

**Figure 6:**
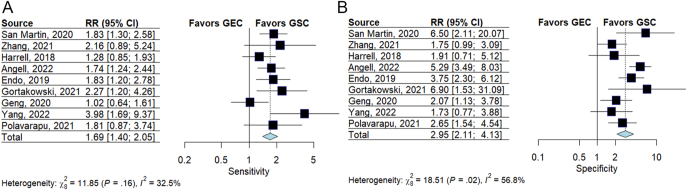
Meta-analysis comparing the sensitivity (A) and specificity (B) of surgically confirmed cases between Afirma GEC and GEC. Squares represent the point estimate for an individual study. The line represents the 95% confidence interval. The red square represents the overall pooled effect.

Nine studies reported surgically confirmed data for both Afirma GSC and GEC (27, 28, 29, 30, 31, 32, 33, 34, 37), eTable 9. A random effects model compared the sensitivity and specificity between the two groups, as seen in Fig. 6. There was a significant difference, indicating that GSC is superior: sensitivity (relative risk: 1.69 (95% CI: 1.40–2.05), specificity (relative risk: 2.95 (95% CI: 2.11–4.13)).

## Discussion

This review assessed the diagnostic accuracy of the Afirma GSC and ThyroSeq v3 molecular tests for detecting thyroid carcinoma in patients with cytologically indeterminate thyroid nodules. The management of indeterminate thyroid nodules poses a clinical challenge, and molecular testing reduces unnecessary diagnostic surgeries.

### Key findings

This review provides an updated synthesis of the literature, including 13 ThyroSeq v3 and 16 Afirma GSC studies, which reflects a larger sample size compared to prior meta-analyses. This review demonstrated that both Afirma GSC and ThyroSeq v3 exhibit high sensitivity (GSC: 0.94 (95% CI: 0.91–0.97); ThyroSeq v3: 0.96 (95% CI: 0.94–0.97)) and a high NPV (GSC: 0.96 (95% CI: 0.89–0.98); ThyroSeq v3: 0.93 (95% CI: 0.85–0.96)). These results demonstrate the value of both tests in ruling out malignancy and reducing unnecessary surgeries. However, both tests have low specificity (Afirma GSC: 0.42 (95% CI: 0.27–0.59); ThyroSeq v3: 0.40 (95% CI: 0.33–0.47)), limiting their reliability in ruling in malignancy. This result is expected, as most molecular tests were developed for exclusion of malignancy, most easily assessed here by NPV. A previous review by Lee *et al.*, analysed six ThyroSeq v3 and seven Afirma GSC studies (16). Their reported sensitivities (0.95 for ThyroSeq V3; 0.96 for Afirma GSC) and specificities (0.50 for ThyroSeq V3; 0.53 for Afirma GSC) align with the findings of this review. Our conclusions are further supported by the comprehensive meta-analysis from Vardali *et al.*, which reported a sensitivity for Afirma GSC and ThyroSeq v3 of 0.97 (0.91–0.99) and 0.95 (0.91–0.97) respectively (49). Their paper reported a specificity for Afirma GSC of 0.50 (0.40–0.61) and for ThyroSeq V3 of 0.47 (0.22–0.73) (49). Our review provides a broader analysis by assessing both surgically confirmed and unconfirmed cases separately, as well as providing an update on the published literature.

### Analysis of unoperated cases (assumed true negatives)

Most benign nodules identified by molecular testing are monitored through clinical follow-up with ultrasound, meaning that relying on surgically confirmed benign nodules does not provide an accurate representation of real-life performance (50). Drawing conclusions based on a small number of test-negative nodules that underwent surgery introduces selection bias. Therefore, it was important for this review to also consider data from cases that remained stable at clinical follow-up. Under this approach, sensitivity increased marginally, but specificity had a greater rise: 0.86 (95% CI: 0.81–0.90) for Afirma GSC and 0.83 (95% CI: 0.79–0.87) for ThyroSeq v3, NPV reached 0.99 (95% CI: 0.98–1.00) and 0.99 (95% CI: 0.98–1.00), respectively. A limitation of this method was the variation in clinical follow-up duration across studies. It is important to note that these values are based on assumption, which has likely resulted in inflation, and the results should be interpreted cautiously. A previous review by Nasr *et al.* applied the same approach for Afirma GSC alone, whereas our review is the first to document this for ThyroSeq v3 (11). In their review, Nasr *et al.* assessed the diagnostic accuracy of Afirma GSC and reported a sensitivity of 0.97 and a negative predictive value (NPV) of 0.995, which closely aligns with the findings for Afirma GSC in our review (11).

### Limitations

The primary limitation of this review is the variability in methodology and reporting across included studies. There was only one randomised controlled trial identified, and there was a lack of direct comparisons of molecular tests within the same cohort, highlighting a need for more robust research.

There is also limited data investigating the impact of molecular test results on quality of life (QoL). Our review identified one study examining Afirma GSC and ThyroSeq v3, drawn from the same cohort as the only randomised controlled trial included (26, 51). Their study reported sustained QoL in patients with a ‘benign’ result, with a poorer QoL in the ‘suspicious’ group, which improved postoperatively. There was no comparison of pre- and post-test QoL. Further studies should focus on the effects of false negatives and false positives on quality of life, and the impact on QoL of avoiding surgery in patients with ‘benign’ results.

A key limitation of this paper and the studies it includes is that the true sensitivity of molecular tests cannot be determined. Most nodules classified as ‘benign’ by a molecular test are not surgically removed and therefore lack histopathological results. Consequently, the observed sensitivity in surgically confirmed cohorts is likely to be overestimated.

## Conclusion

Both Afirma GSC and ThyroSeq v3 are effective tools for managing indeterminate thyroid nodules, primarily by ruling out malignancy and reducing unnecessary diagnostic surgeries. Limited evidence supports the superiority of one test over the other, and both remain appropriate diagnostic tests. Future research should prioritise randomised controlled trials, long-term follow-up of unoperated nodules, and direct comparisons of molecular tests to assess the impact on patients.

## Supplementary materials



## Declaration of interest

There is no conflict of interest that could be perceived as prejudicing the impartiality of the research reported.

## Funding

This research did not receive any specific grant from any funding agency in the public, commercial, or not-for-profit sector.

## Author contribution statement

ND contributed to the design of the work, first reviewer, literature searches, data extraction, and writing of the paper. SB was responsible for literature searches, second reviewer, and data extraction. JM helped in revision of text, critical appraisal, and helped draft and edit writing. KB critically appraised data and writing, and helped draft writing. HN supervised the project, critically appraised data, revised text, and suggested the study question.
