# Supplementary material for: Comparing the diagnostic accuracy of Afirma GSC to ThyroSeq V3 in cytologically indeterminate thyroid nodules
Source: Eur Thyroid J. 2025 Dec 9;14(6):e250296. doi: 10.1530/ETJ-25-0296 (PMC12697243; doi:10.1530/ETJ-25-0296)
Supplement: Supplementary file 1 [file supplementary_materials.pdf]

Supplementary material:

**eTable 1: Systematic Search Strategy**			
**#**	**Database**	**Search Terms**	
1	PubMed	("Indeterminate"[Title/Abstract] OR "Thy3"[Title/Abstract] OR "Thy4"[Title/Abstract] OR "bethesda category 3"[Title/Abstract] OR "bethesda category 4"[Title/Abstract] OR "bethesda category iii"[Title/Abstract] OR "bethesda category iv"[Title/Abstract]) AND ("ThyroSeq"[Title/Abstract] OR "Afirma"[Title/Abstract] OR "Affirma"[Title/Abstract] OR "GSC"[Title/Abstract] OR "molecular diagnostic"[Title/Abstract] OR "genetic testing"[Title/Abstract] OR "molecular marker"[Title/Abstract] OR "genom*"[Title/Abstract] OR "ThyGenX"[Title/Abstract] OR "ThyGeNEXT"[Title/Abstract] OR "ThyraMIR"[Title/Abstract] OR "Rosetta"[Title/Abstract] OR "ThyroSpec"[Title/Abstract] OR "miRInform"[Title/Abstract] OR "AmpliSeq"[Title/Abstract] OR "mir-THYpe"[Title/Abstract] OR "Gene sequencing classifier"[Title/Abstract] OR "Next generation sequencing"[Title/Abstract] OR "cytopathology"[Title/Abstract]) AND ("thyroid gland"[Title/Abstract] OR "thyroid nodule"[Title/Abstract] OR "thyroid neoplasms"[Title/Abstract] OR "thyroid cancer"[Title/Abstract] OR "thyroid carcinoma"[Title/Abstract] OR "thyroid microcarcinoma"[Title/Abstract] OR "thyroid tum*"[Title/Abstract] OR "thyroid adenoma"[Title/Abstract] OR "thyroid adenocarcinoma"[Title/Abstract] OR "thyroid nod*"[Title/Abstract] OR "thyroid lump"[Title/Abstract] OR "thyroid swelling"[Title/Abstract] OR "Follicular"[Title/Abstract] OR "papillary"[Title/Abstract] OR "malignant"[Title/Abstract]) AND ("biopsy, fine needle"[MeSH Terms] OR ("biopsy"[All Fields] AND "fine needle"[All Fields]) OR "fine-needle biopsy"[All Fields] OR ("fine"[All Fields] AND "needle"[All Fields] AND "aspiration"[All Fields]) OR "fine needle aspiration"[All Fields] OR ("biopsy, fine needle"[MeSH Terms] OR ("biopsy"[All Fields] AND "fine needle"[All Fields]) OR "fine-needle biopsy"[All Fields] OR ("fine"[All Fields] AND "needle"[All Fields] AND "biopsy"[All Fields]) OR "fine needle biopsy"[All Fields]))	687
2	Embase and Ovid-Medline	("Indeterminate": ti.ab OR "Thy3":ti.ab OR "Thy4":ti.ab OR "Bethesda category 3":ti.ab OR "Bethesda category 4":ti.ab OR "Bethesda category iii" :ti.ab OR "Bethesda category iv" :ti.ab) AND ("ThyroSeq":ti.ab OR "Afirma": ti.ab OR "Affirma":ti.ab OR "GSC" :ti.ab OR "molecular diagnostic":ti.ab OR "genetic testing" :ti.ab OR "molecular marker" :ti.ab OR "genom*":ti.ab OR "ThyGenX":ti.ab OR "ThyGeNEXT":ti.ab OR "ThyraMIR" :ti.ab OR "Rosetta" :ti.ab OR "ThyroSpec":ti.ab OR "miRInform" :ti.ab OR "AmpliSeq" :ti.ab OR "mir-THYpe":ti.ab OR "Gene sequencing classifier" :ti.ab OR "Next generation sequencing" :ti.ab OR "cytopathology" :ti.ab) AND ("thyroid gland" :ti.ab OR "thyroid nodule":ti.ab OR "thyroid neoplasms":ti.ab OR "thyroid cancer":ti.ab OR "thyroid carcinoma":ti.ab OR "thyroid microcarcinoma":ti.ab OR "thyroid tum*":ti.ab OR "thyroid adenoma" :ti.ab OR "thyroid adenocarcinoma" :ti.ab OR "thyroid nod*" :ti.ab OR "thyroid lump" :ti.ab OR "thyroid swelling" :ti.ab OR "Follicular" :ti.ab OR "papillary" :ti.ab OR "malignant" :ti.ab) AND ("fine needle biopsy": af))	687/341
3	Cochrane Library	#1 Thyroid Cancer 2584<br>#2 MeSH descriptor: [Thyroid Nodule] explode all trees226<br>#3 MeSH descriptor: [Thyroid Gland] explode all trees 705<br>#4 MeSH descriptor: [Thyroid Neoplasms] explode all trees 1035<br>#5 Thyroid carcinoma 1226<br>#6 Thyroid microcarcinoma 48<br>#7 Thyroid tumour 1080<br>#8 Thyroid adenoma 147	2

		#9 Thyroid adenocarcinoma 145<br>#10 Thyroid node 436<br>#11 Thyroid lump 6<br>#12 Thyroid swelling 148<br>#13 Follicular 6466<br>#14 Papillary 2356<br>#15 Malignant 24590<br>#16 ThyroSeq 10<br>#17 Afirma 9<br>#18 GSC 146<br>#19 Molecular diagnostic 3130<br>#20 Genetic testing 4577<br>#21 Molecular marker 1631<br>#22 Genome 4568<br>#23 ThyGenX 0<br>#24 ThyGeNEXT 0<br>#25 ThyraMIR 0<br>#26 Rosetta 46<br>#27 miRInform 1<br>#28 AmpliSeq 40<br>#29 mir-THYpe 0<br>#30 Gene sequencing classifier 55<br>#31 "Next generation Sequencing" 1645<br>#32 Cytopathology 273<br>#33 Indeterminate 1106<br>#34 Thy3 2<br>#35 Bethesda category 3 OR Bethesda category 4 OR Bethesda category III OR Bethesda category IV 75<br>#36 MeSH descriptor: [Biopsy, Fine-Needle] explode all trees 407<br>#37 #1 OR #2 OR #3 OR #4 OR #5 OR #6 OR #7 OR #8 OR #9 OR #10 OR #11 OR #12 OR #13 OR #14 OR #15 35868<br>#38 #16 OR #17 OR #18 OR #19 OR #20 OR #21 OR #22 OR #23 OR #24 OR #25 OR #26 OR #27 OR #28 OR #29 OR #30 OR #31 OR #32 OR #33 15676<br>#39 #34 OR #35 77<br>#40 #36 AND #37 AND #38 AND #39 2	
4	Web of Science	1: (((((((((((((AB=(thyroid gland)) OR AB=(Thyroid Nodule)) OR AB=(Thyroid Neoplasms)) OR AB=(Thyroid Cancer)) OR AB=(Thyroid carcinoma*)) OR AB=(Thyroid microcarcinoma*)) OR AB=(Thyroid Tum*)) OR AB=(Thyroid adenoma)) OR AB=(Thyroid Adenocarcinoma)) OR AB=(Thyroid nod*)) OR AB=(Thyroid lump)) OR AB=(Thyroid swelling)) OR AB=(Follicular)) OR AB=(Papillary)) OR AB=(Malignant): 543,997<br><br>2: ((((((((((((((AB=(ThyroSeq)) OR AB=(Afirma)) OR AB=(GSC)) OR AB=(Molecular diagnostic*)) OR AB=(Genetic testing)) OR AB=(Molecular marker)) OR AB=(Genom*)) OR AB=(ThyGenX)) OR AB=(ThyGeNEXT)) OR AB=(ThyraMIR)) OR AB=(Rosetta)) OR AB=(ThyroSpec)) OR AB=(miRInform)) OR AB=(AmpliSeq)) OR AB=(Gene sequencing classifier)) OR AB=(mir-THYpe): 1,283,550	149

		3: ((AB=(Indeterminate)) OR AB=("Thy3*")) OR AB=(Bethesda category 3 or Bethesda category 4 or Bethesda category III or Bethesda category IV): 26,896<br><br>4: AB=(Fine needle biopsy): 14,813<br><br>5: #1 AND #2 AND #4 Results: 149	

eTable 2: Study characteristics

Author	Year	Title	Study Design	Country	Length of follow up (months)	Setting	Molecular test
		**Afirma**					
Livhits et al (1)	2020	Effectiveness of Molecular Testing Techniques for Diagnosis of Indeterminate Thyroid Nodules A Randomised Clinical Trial	RCT	USA	Median: 12.2 (8.1-17.9)	Multi-institute	Afirma GSC/ ThyroSeq V3
Patel et al (2)	2018	Performance of a Genomic Sequencing Classifier for the Preoperative Diagnosis of Cytologically Indeterminate Thyroid Nodules	Prospective blinded study	USA	Limited data	Multi-institute	Afirma GSC
Angell et al (3)	2019	Independent Comparison of the Afirma Genomic Sequencing Classifier and Gene Expression Classifier for Cytologically Indeterminate Thyroid Nodules	Retrospective	USA	Limited data	Single institute	Afirma GSC and GEC
Endo et al (4)	2019	Afirma Gene Sequencing Classifier Compared with Gene Expression Classifier in Indeterminate Thyroid Nodules	Retrospective analysis	USA	Median: 7.5	Single institute	Afirma GSC and GEC
Harrell et al (5)	2019	Statistical comparison of Afirma GSC and Afirma GEC outcomes in a community endocrine surgical practice: Early findings	retrospective	USA	Limited data	Single institute	Afirma GSC and GEC

San Martin et al (6)	2020	Real-world Comparison of Afirma GEC and GSC for the Assessment of Cytologically Indeterminate Thyroid Nodules	Retrospective analysis	USA	12 months	Single institute	GSC and GEC
Geng et al (7)	2020	Comparison of Afirma Gene Expression Classifier with Gene Sequencing Classifier in indeterminate thyroid nodules: A single-institutional experience	retrospective	USA	6 to 41 months	Single institute	Afirma GSC and GEC
Polavarapu et al (8)	2021	Comparison of Afirma GEC and GSC to Nodules Without Molecular Testing in Cytologically Indeterminate Thyroid Nodules	Retrospective	USA	Mean 20.6 (SD: 12)	Multi centre	GSC and GEC
Zhang et al (9)	2021	Performance of Afirma genomic sequencing classifier vs gene expression classifier in Bethesda category III thyroid nodules: An institutional experience	retrospective	USA	6 months	Single institute	Afirma GSC and GEC
Gortakowski et al (10)	2021	Single institution experience with Afirma and ThyroSeq testing in indeterminate thyroid nodules	retrospective DTA	USA	US at 1 year: TN	Single institute	GSC/ ThyroSeq V3
Jin et al (a) (11)	2023	Performance of Afirma genomic sequencing classifier and histopathological outcome in Bethesda category III thyroid nodules: Initial versus repeat fine-needle aspiration	Retrospective	USA	minimum 6 months, mean 21 months (6-63)	Single centre	GSC
Jin et al (b) (12)	2022	Performance of Afirma genomic sequencing classifier and histopathological outcome are associated with patterns of atypia in Bethesda category III thyroid nodules	Retrospective cohort	USA	minimum 6 months	Single Centre	GSC
Yang et al (13)	2022	Performance of Afirma Gene Sequencing Classifier versus Gene Expression Classifier in thyroid nodules with indeterminate cytology	Retrospective	USA	minimum 6 months	Single institute	Afirma GSC, Afirma GEC

Loncar et al (14)	2023	European experience with the Afirma Gene Expression Classifier for indeterminate thyroid nodules: A clinical utility study in the Netherlands	Prospective	Netherlands	65 months (62.5-68 months)	Multi institute	GSC
Ahmadi et al (15)	2024	Outcomes of Cytologically Indeterminate Thyroid Nodules Managed With Genomic Sequencing Classifier	Retrospective Cohort	USA	Median: 23 months	Multi institute	Afirma GSC
Kim et al (16)	2023	Bethesda III and IV Thyroid Nodules Managed Nonoperatively After Molecular Testing With Afirma GSC or ThyroSeq v3	Prospective follow up of randomised clinical trial	USA	Median: 34 (12-60)	Single institute	Afirma GSC, ThyroSeq V3
		ThyroSeq:					
Steward et al (17)	2018	Performance of a Multigene Genomic Classifier in Thyroid Nodules with Indeterminate Cytology: A Prospective Blinded Multicentre Study	Prospective double-blinded	USA and Singapore	Excluded patients who did not have surgery	Multi institute	ThyroSeq V3
Jug et al (18)	2020	High-risk and intermediate-high–risk results from the ThyroSeq v2 and v3 thyroid genomic classifier are associated with neoplasia: Independent performance assessment at an academic institution	Retrospective analysis	USA	Limited data	Single institute	ThyroSeq V3
Carty et al (19)	2020	The Clinical Utility of Molecular Testing in the Management of Thyroid Follicular Neoplasms (Bethesda IV Nodules)	Retrospective analysis	USA	Mean 24.6 (5.3-62.5)	Single institute	ThyroSeq V3

Chen et al (20)	2019	The Role of the ThyroSeq v3 Molecular Test in the Surgical Management of Thyroid Nodules in the Canadian Public Health Care Setting	Prospective	Canada	Limited data	Single institute	ThyroSeq V3
Desai et al (21)	2021	ThyroSeq v3 for Bethesda III and IV: An institutional experience	Retrospective	USA	assumed	Single institute	ThyroSeq V3
Gajzer et al (22)	2022	Probability of malignancy as determined by ThyroSeq v3 genomic classifier varies according to the subtype of atypia	Retrospective	USA	surgically confirmed	Single institute	ThyroSeq V3
Tjendra et al (23)	2023	Probability of malignancy and molecular alterations as determined by ThyroSeq v3 genomic classifier in Bethesda Category IV	Retrospective	USA	> 9 months	Single institute	ThyroSeq V3
Sirotnikov et al (24)	2024	ThyroSeq overview on indeterminate thyroid nodules: An institutional experience	Retrospective cohort	USA	6 to 18 months	Single-Institute	ThyroSeq V3
Yang et al (25)	2024	Performance of a multigene genomic classifier and clinical parameters in predicting malignancy in a Southeast Asian cohort of patients with cytologically indeterminate thyroid nodules	Prospective	Singapore	Limited data	Multi institute	ThyroSeq V3
Lévesque et al (26)	2025	Publicly Funded Molecular Testing of Indeterminate Thyroid Nodules: Canada's Experience	Prospective	Canada	2 years	Multi institute	Thyroseq V3

eTable 3: Patient Characteristics

Authors	Year	Patient selection method	Mean Age	Sex	
				Male	Female
**Afirma**					
Livhits et al (1)	2020	Consecutive 2017 to 2019	Median: 55 (IQR: 44-67)	80 (23.1%)	266 (76.9)
Patel et al (2)	2018	Consecutive 2009 to 2010	51.7 (22-85)	41 (22.4%)	142 (77.6%)
Angell et al (3)	2019	Consecutive: GEC (2011-2017), GSC (2017-2018)	GEC: 56 (21-87), GSC: 57 (20-87)	GEC: 92 (20.3%), GSC: 17 (15.55)	GEC: 361 (79.7%). GSC: 93 (84.55)
Endo et al (4)	2019	Consecutive 2011 to 2017	GEC: 53.0 (14.3). GSC: 51.4 (14.9)	GEC: 36 (21.8), GSC: 41 (33)	GEC: 137 (77.0), GSC: 129 (78.2)
Harrell et al (5)	2019	Consecutive 2011 to 2018	(no data)	(no data)	(no data)
San Martin et al (6)	2020	GSC: Consecutive 2017 to 2018. GEC: 2011 to 2017	GSC: 56.1 ± 12.7, GEC: 58.6 ± 15.6	GSC: 29 (15%). GEC: 64 (36.8%)	GSC: 87 (75%). GEC: 110 (63.2%)
Geng et al (7)	2020	Consecutive: I GEC: 2015-2017. GSC: 2017 to 2019	Median: 53 (20-83)	no data	no data
Polavarapu et al (8)	2021	Consecutive: GEC: 2013-2017, GSC: 2017-2019	GEC: 55.4 ± 16.7, GSC: 56.17 ± 15.7	GEC: 26 (37), GSC: 39 (31)	GEC: 45 (63), GSC: 85 (69)
Zhang et al (9)	2021	Consecutive 2013 to 2020	GSC: Median: 47, 19-83. GEC: 55 (18-90)	GSC: 33 (26.4%). GEC: 32 (26.7%)	GSC: 92 (73.6%). GEC: 88 (73.3%)
Gortakowski et al (10)	2021	consecutive: 2014 to 2020	GSC: 56.4, TS3: 55.3. GEC: 56.6	GSC: 12 (17), TS3: 12 (22). GEC: 26 (29)	GSC: 58 (83), TS3: 43(78) GEC: 63 (71)
Jin et al (a) (11)	2023	consecutive: 2017 to 2021	52 (30-77)	14	53

Jin et al (b) (12)	2022	Consecutive: 2017-2021	55 (19–82)	45	90
Yang et al (13)	2022	Consecutive: GEC: 2012- 2017. GSC: 2017 to 2021	Mean: GEC: 51.7 ± 16.3 GSC: 52.2 ± 15.7	GEC: 13 (27), GSC: 12 (24)	GEC: 36 (73), GSC: 39 (76)
Loncar et al (14)	2023	Consecutive: 2016-2018	GEC: 52.5 ± 12.2	19 (28.8%)	GEC: 47 (71.2),
Ahmadi et al (15)	2024	Consecutive: 2017-2021	Median: 59	125 (15%)	709 (75%)
Kim et al (16)	2023	consecutive 2017-2019	Median: 56 (IQR: 44-67)	All: 43 (22%)	All: 151 (78%)
ThyroSeq					
Steward et al (17)	2018	Consecutive 2015 to 2016	Median: 53 (18-90)	20%	202 (79%)
Jug et al (18)	2020	Over 2 years	ThyroSeq V2: 58.14 [9-87]. V3: 58.98 [14-85]	ThyroSeq V2: 18 (20), V3: 17 (19)	ThyroSeq v2: 72 (80), V3: 71 (81)
Carty et al (19)	2020	Consecutive: V2: 2014 to 2017, V3: 2017-2019	54 years (±15.6)	27%	74.50%
Chen et al (20)	2019	Consecutive 2019	54	37	13
Desai et al (21)	2021	Consecutive 2017 to 2019	(no data)	(no data)	(no data)
Gajzer et al (22)	2022	Consecutive 2018 o 2020	(no data)	(no data)	(no data)
Tjendra et al (23)	2023	Consecutive 2018 to 2022	(-)	(-)	(-)
Sirotnikov et al (24)	2024	Consecutive (2017-2019)	55.2 (17 to 90)	125 (19%)	533 (81%)
Yang et al (25)	2024	Consecutive 2016 to 2019	Mean: 52.2 ± 15.7	33 (25%)	99 (75%)
Lévesque et al (26)	2025	Consecutive 2021 to 2022	TSv3 negative: 59.0 (49.0-67.0), TSv3 positive: 55.0 (43.0-65.0)	94 (18.8%)	406 (81.2%)

Author, year	Reporting	External validity	Internal validity-bias	Internal validity-confounding	Power	Total Score	Quality category
Livhits et al, 2021 (1)	9	3	3	5	1	22	Good
Patel et al, 2018 (2)	9	3	4	3	0	20	Good
Angell et al, 2022 (3)	9	3	3	2	0	17	Fair
Endo et al, 2019 (4)	9	3	4	2	0	19	Fair
Harrell et al, 2018 (5)	8	3	2	2	0	12	Poor
San Martin et al, 2019 (6)	9	3	3	2	0	18	Fair
Geng et al, 2020 (7)	4	3	3	2	0	13	Poor
Polavarapu et al, 2021 (8)	9	3	3	2	0	18	Fair
Zhang et al, 2021 (9)	4	3	3	2	0	16	Fair
Gortakowski et al, 2021 (10)	9	3	3	2	0	19	Fair
Jin et al, 2023 (11)	7	3	3	2	0	17	Fair
Jin et al, 2022 (12)	10	3	3	2	0	17	Fair
Yang et al, 2022 (13)	8	3	3	2	0	18	Fair
Loncar et al, 2023 (14)	8	3	4	3	0	21	Good
Ahmadi et al, 2024 (15)	10	3	4	3	0	20	Good
Kim et al, 2023 (16)	9	3	3	4	0	20	Good
Steward et al, 2018 (17)	10	3	4	2	0	18	Fair
Jug et al, 2020 (18)	9	3	3	2	0	16	Fair
Carty et al, 2020 (19)	8	3	3	2	0	18	Fair
Chen et al, 2020 (20)	7	3	3	2	0	16	Fair
Desai et al, 2020 (21)	9	3	3	2	0	18	Fair
Gajzer et al (22)	7	3	3	2	0	14	Poor
Tjendra et al, 2023 (23)	9	3	3	2	0	14	Poor
Sirotnikov et al, 2024 (24)	5	3	3	3	0	19	Fair

Yang et al, 2024 (25)	5	3	4	2	0	19	Fair
Lévesque et al, 2025 (26)	9	3	4	2	0	18	Fair

eTable 4: Quality Assessment Table

eTable 5: Outcome data for studies containing surgical outcome data for Afirma GSC

**Author**	**Year**	**Molecular test**	**Nodules (surgically confirmed)**	**Sensitivity**	**Specificity**	**TP**	**FP**	**FN**	**TN**	**NPV**	**PPV:**
Livhits et al (1)	2020	GSC	70	1.00	0.31	31 (44%)	27 (39%)	0	12 (17%)	1.00	0.53
Patel et al (2)	2018	GSC	190	0.91	0.68	41 (22%)	46 (24%)	4 (2%)	99 (52%)	0.96	0.47
Angell et al (3)	2019	GSC	44	0.96	0.20	23 (52%)	16 (37%)	1 (2%)	4 (9%)	0.80	0.59
Endo et al (4)	2019	GSC	29	1.00	0.29	15 (52%)	10 (34%)	0	4 (14%)	1.00	0.60
Harrell et al (5)	2019	GSC	45	0.97	0.44	28 (62%)	9 (20%)	1 (2%)	7 (16%)	0.88	0.76
San Martin et al (6)	2019	GSC	42	0.91	0.50	29 (69%)	5 (12%)	3 (7%)	5 (12%)	0.63	0.85
Geng et al (7)	2020	GSC	41	1.00	0.42	17 (41%)	14 (34%)	0	10 (24%)	1.00	0.55
Polavarapu et al (8)	2021	GSC	47	0.94	0.17	17 (36%)	24 (51%)	1 (2%)	5 (11%)	0.83	0.41
Zhang et al (9)	2021	GSC	43	1.00	0.42	10 (23%)	19 (44%)	0	14 (33%)	1.00	0.34
Gortakowski et al (10)	2021	GSC	15	1.00	0.40	10 (67%)	3 (20%)	0	2 (13%)	1.00	0.77
Jin et al (a) (11)	2023	GSC	64	1.00	0.41	13 (20%)	30 (47%)	0	21 (33%)	1.00	0.30
Jin et al (b) (12)	2022	GSC	61	1.00	0.38	13 (21%)	30 (49%)	0	18 (30%)	1.00	0.30
Yang et al (13)	2022	GSC	21	1.00	0.58	9 (43%)	5 (24%)	0	7 (33%)	1.00	0.64
Ahmadi et al (15)	2024	GSC	329	0.95	0.33	143 (43%)	119 (36%)	8 (2%)	59 (18%)	0.88	0.55
Kim et al (16)	2023	GSC	81	1.00	0.40	36 (44%)	27 (33%)	0	18 (22%)	1.00	0.57

eTable 6: Assuming unoperated test negative are true negative: Afirma GSC

Author	Year	Molecular test	Nodules (assumed)	Sensitivity	Specificity	TP	FP	FN	TN	NPV	PPV:
Livhits et al (1)	2020	GSC	163	1.00	0.80	31 (19%)	27 (17%)	0	105 (64%)	1.00	0.53
Angell et al (3)	2019	GSC	114	0.96	0.82	23 (20%)	16 (14%)	1 (1%)	74 (65%)	0.99	0.59
Endo et al (4)	2019	GSC	150	1.00	0.93	15 (10%)	10 (7%)	0	125 (83%)	1.00	0.60
Harrell et al (5)	2019	GSC	122	0.97	0.90	28 (23%)	9 (7%)	1 (1%)	84 (69%)	0.99	0.76
San Martin et al (6)	2019	GSC	116	0.91	0.94	29 (25%)	5 (4%)	3 (3%)	79 (68%)	0.96	0.85
Geng et al (7)	2020	GSC	112	1.00	0.85	17 (15%)	14 (13%)	0	81 (72%)	1.00	0.55
Polavarapu et al (8)	2021	GSC	116	0.94	0.76	17 (15%)	24 (21%)	1 (1%)	74 (64%)	0.99	0.41
Zhang et al (9)	2021	GSC	135	1.00	0.84	9 (7%)	20 (15%)	0	106 (79%)	1.00	0.31
Gortakowski et al (10)	2021	GSC	70	1.00	0.95	10 (14%)	3 (4%)	0	57 (81%)	1.00	0.77
Jin et al (a) (11)	2023	GSC	133	1.00	0.74	11 (8%)	32 (24%)	0	90 (68%)	1.00	0.26
Jin et al (b) (12)	2022	GSC	133	1.00	0.75	13 (10%)	30 (23%)	0	90 (68%)	1.00	0.30
Yang et al (13)	2022	GSC	46	1.00	0.86	9 (20%)	5 (11%)	0	32 (70%)	1.00	0.64
Loncar et al (14)	2023	GSC	64	0.63	0.71	10 (16%)	14 (22%)	6 (9%)	34 (53%)	0.85	0.42
Ahmadi et al (15)	2024	GSC	790	0.95	0.81	143 (18%)	119 (15%)	8 (1%)	520 (66%)	0.98	0.55
Kim et al (16)	2023	GSC	152	1.00	0.77	36 (24%)	27 (18%)	0	89 (59%)	1.00	0.57

eTable 7: Surgically confirmed outcome data for ThyroSeq V3.

Author	Year	Molecular test	Nodules: surgically confirmed	Sensitivity	Specificity	TP	FP	FN	TN	NPV:	PPV:
Steward et al (17)	2019	TSV3	247	0.94	0.82	64 (26%)	33 (13%)	4 (2%)	146 (59%)	0.97	0.66
Jug et al (18)	2020	TSV3	27	1.00	0.17	9 (33%)	15 (56%)	0	3 (11%)	1.00	0.38
Carty et al (19)	2020	TSV3	53	0.97	0.35	35 (66%)	11 (21%)	1 (2%)	6 (11%)	0.86	0.76
Chen et al (20)	2020	TSV3	22	0.95	1.00	19 (86%)	0	1 (5%)	2 (9%)	0.67	1.00
Gortakowski et al (10)	2021	TSV3	19	0.80	0.14	4 (21%)	12 (63%)	1 (5%)	2 (11%)	0.67	0.25
Desai et al (21)	2021	TSV3	127	0.93	0.46	65 (51%)	31 (24%)	5 (4%)	26 (20%)	0.84	0.68
Livhits et al (1)	2021	TSV3	60	0.97	0.36	31 (52%)	18 (30%)	1 (2%)	10 (17%)	0.91	0.63
Gajzer et al (22)	2022	TSV3	155	0.97	0.47	62 (40%)	48 (31%)	2 (1%)	43 (28%)	0.96	0.56
Kim et al (16)	2023	TSV3	63	0.97	0.40	32 (51%)	18 (29%)	1 (2%)	12 (19%)	0.92	0.64
Tjendra et al (23)	2023	TSV3	52	0.87	0.38	20 (38%)	18 (35%)	3 (6%)	11 (21%)	0.79	0.53
Sirotnikov et al (24)	2024	TSV3	84	0.86	0.65	43 (51%)	12 (14%)	7 (8%)	22 (26%)	0.76	0.78
Yang et al (25)	2024	TSV3	109	0.87	0.72	48 (44%)	15 (14%)	7 (6%)	39 (36%)	0.85	0.76
Lévesque et al (26)	2025	TSV3	109	1.00	0.03	73 (67%)	35 (32%)	0	1 (1%)	1.00	0.68

eTable 8: Assuming non operated test negative results are true negatives: ThyroSeq V3

Author	Year	Molecular test	Nodules: assuming benign	Sensitivity	Specificity	TP	FP	FN	TN	NPV:	PPV:
Jug et al (18)	2020	TSV3	86	1.00	0.81	9 (10%)	15 (17%)	0	62 (72%)	1.00	0.38
Chen et al (20)	2020	TSV3	49	0.95	1.00	19 (39%)	0	1 (2%)	29 (59%)	0.97	1.00
Gortakowski et al (10)	2021	TSV3	56	0.80	0.76	4 (7%)	12 (21%)	1 (2%)	39 (70%)	0.98	0.25
Desai et al (21)	2021	TSV3	390	0.93	0.90	65 (17%)	31 (8%)	5 (1%)	289 (74%)	0.98	0.68
Livhits et al (1)	2021	TSV3	150	0.97	0.85	31 (21%)	18 (12%)	1 (1%)	100 (67%)	0.99	0.63
Gajzer et al (22)	2022	TSV3	542	0.97	0.90	62 (11%)	48 (9%)	2 (~0%)	430 (79%)	1.00	0.56
Kim et al (16)	2023	TSV3	137	0.97	0.83	32 (23%)	18 (13%)	1 (1%)	86 (63%)	0.99	0.64
Tjendra et al (23)	2023	TSV3	83	0.87	0.70	20 (24%)	18 (22%)	3 (4%)	42 (51%)	0.93	0.53
Lévesque et al (26)	2025	TSV3	471	1.00	0.91	73 (16%)	35 (7%)	0	363 (77%)	1.00	0.68

eTable 9: Outcome data from studies containing data for both Afirma GEC and Afirma GSC

Author	Year	GEC							GSC						
		**Nodules**	**Sensitivity**	**Specificity**	**TP**	**FP**	**FN**	**TN**	**Nodules**	**Sensitivity**	**Specificity**	**TP**	**FP**	**FN**	**TN**
San Martin et al (6)	2020	85	0.97	0.08	32	48	1	4	42	0.91	0.50	29	5	3	5
Zhang et al (9)	2021	65	1.00	0.24	7	44	0	14	43	1.00	0.42	10	19	0	14
Harrell et al (5)	2018	35	0.94	0.24	17	13	1	4	45	0.97	0.44	28	9	1	7
Angell et al (3)	2022	249	0.97	0.15	75	146	2	26	44	0.96	0.20	23	16	1	4
Endo et al (4)	2019	180	0.94	0.19	51	102	3	24	29	1.00	0.29	15	10	0	4
Gortakowski et al (10)	2021	34	0.91	0.09	10	21	1	2	15	1.00	0.40	10	3	0	2
Geng et al (7)	2020	71	0.91	0.28	29	28	3	11	41	1.00	0.42	17	14	0	10
Yang et al (13)	2022	65	1.00	0.24	7	44	0	14	21	1.00	0.58	9	5	0	7
Polavarapu et al (8)	2021	40	1.00	0.31	8	22	0	10	47	0.94	0.17	17	24	1	5

eReferences:

1. Livhits MJ, Zhu CY, Kuo EJ, Nguyen DT, Kim J, Tseng CH, Leung AM, Rao J, Levin M, Douek ML, Beckett KR, Cheung DS, Gofnung YA, Smooke-Praw S, Yeh MW. Effectiveness of Molecular Testing Techniques for Diagnosis of Indeterminate Thyroid Nodules: A Randomized Clinical Trial. JAMA Oncol. 2021 Jan 1;7(1):70-77. doi: 10.1001/jamaoncol.2020.5935. PMID: 33300952; PMCID: PMC7729582.
2. Patel KN, Angell TE, Babiarz J, Barth NM, Blevins T, Duh QY, Ghossein RA, Harrell RM, Huang J, Kennedy GC, Kim SY, Kloos RT, LiVolsi VA, Randolph GW, Sadow PM, Shanik MH, Sosa JA, Traweek ST, Walsh PS, Whitney D, Yeh MW, Ladenson PW. Performance of a Genomic Sequencing Classifier for the Preoperative Diagnosis of Cytologically Indeterminate Thyroid Nodules. JAMA Surg. 2018 Sep 1;153(9):817-824. doi: 10.1001/jamasurg.2018.1153. PMID: 29799911; PMCID: PMC6583881.
3. Angell TE, Heller HT, Cibas ES, Barletta JA, Kim MI, Krane JF, Marqusee E. Independent Comparison of the Afirma Genomic Sequencing Classifier and Gene Expression Classifier for Cytologically Indeterminate Thyroid Nodules. Thyroid. 2019 May;29(5):650-656. doi: 10.1089/thy.2018.0726. Epub 2019 Mar 22. PMID: 30803388.
4. Endo M, Nabhan F, Porter K, Roll K, Shirley LA, Azaryan I, Tonkovich D, Perlick J, Ryan LE, Khawaja R, Meng S, Phay JE, Ringel MD, Sipos JA. Afirma Gene Sequencing Classifier Compared with Gene Expression Classifier in Indeterminate Thyroid Nodules. Thyroid. 2019 Aug;29(8):1115-1124. doi: 10.1089/thy.2018.0733. Epub 2019 Jul 17. PMID: 31154940; PMCID: PMC7141558.
5. Harrell RM, Eyerly-Webb SA, Golding AC, Edwards CM, Bimston DN. Statistical comparison of Afirma GSC and Afirma GEC outcomes in a community endocrine surgical practice: Early findings. Endocr Pract. 2019 Feb;25(2):161-164. doi: 10.4158/EP-2018-0395. Epub 2018 Nov 1. PMID: 30383497.
6. San Martin VT, Lawrence L, Bena J, Madhun NZ, Berber E, Elsheikh TM, Nasr CE. Real-world Comparison of Afirma GEC and GSC for the Assessment of Cytologically Indeterminate Thyroid Nodules. J Clin Endocrinol Metab. 2020 Mar 1;105(3):dgz099. doi: 10.1210/clinem/dgz099. PMID: 31665322.
7. Geng Y, Aguilar-Jakthong JS, Moatamed NA. Comparison of Afirma Gene Expression Classifier with Gene Sequencing Classifier in indeterminate thyroid nodules: A single-institutional experience. Cytopathology. 2021 Mar;32(2):187-191. doi: 10.1111/cyt.12920. Epub 2020 Dec 5. PMID: 33010060.
8. Polavarapu P, Fingeret A, Yuil-Valdes A, Olson D, Patel A, Shivaswamy V, Matthias TD, Goldner W. Comparison of Afirma GEC and GSC to Nodules Without Molecular Testing in Cytologically Indeterminate Thyroid Nodules. J Endocr Soc. 2021 Oct 7;5(11):bvab148. doi: 10.1210/jendso/bvab148. PMID: 34708178; PMCID: PMC8543699.
9. Zhang L, Smola B, Lew M, Pang J, Cantley R, Pantanowitz L, Heider A, Jing X. Performance of Afirma genomic sequencing classifier vs gene expression classifier in Bethesda category III thyroid nodules: An institutional experience. Diagn Cytopathol. 2021 Aug;49(8):921-927. doi: 10.1002/dc.24765. Epub 2021 May 22. PMID: 34021740.
10. Gortakowski M, Feghali K, Osakwe I. Single Institution Experience with Afirma and Thyroseq Testing in Indeterminate Thyroid Nodules. Thyroid. 2021 Sep;31(9):1376-1382. doi: 10.1089/thy.2020.0801. Epub 2021 Apr 29. PMID: 33764195.

11. Jin X, Lew M, Pantanowitz L, Iyengar JJ, Haymart MR, Papaleontiou M, Broome D, Sandouk Z, Raja SS, Hughes DT, Smola B, Jing X. Performance of Afirma genomic sequencing classifier and histopathological outcome in Bethesda category III thyroid nodules: Initial versus repeat fine-needle aspiration. Diagn Cytopathol. 2023 Nov;51(11):698-704. doi: 10.1002/dc.25203. Epub 2023 Jul 31. PMID: 37519144.
12. Jin X, Lew M, Pantanowitz L, Smola B, Jing X. Performance of Afirma genomic sequencing classifier and histopathological outcome are associated with patterns of atypia in Bethesda category III thyroid nodules. Cancer Cytopathol. 2022 Nov;130(11):891-898. doi: 10.1002/cncy.22625. Epub 2022 Jul 5. PMID: 35789120; PMCID: PMC9796557.
13. Yang Z, Zhang T, Layfield L, Esebua M. Performance of Afirma Gene Sequencing Classifier versus Gene Expression Classifier in thyroid nodules with indeterminate cytology. J Am Soc Cytopathol. 2022 Mar-Apr;11(2):74-78. doi: 10.1016/j.jasc.2021.07.002. Epub 2021 Jul 22. Erratum in: J Am Soc Cytopathol. 2024 Nov-Dec;13(6):458. doi: 10.1016/j.jasc.2024.07.002. PMID: 34366280.
14. Lončar I, van Velsen EFS, Massolt ET, van Kemenade FJ, van Engen-van Grunsven ACH, van Hemel BM, van Nederveen FH, Netea-Maier R, Links TP, Peeters RP, van Ginhoven TM. European experience with the Afirma Gene Expression Classifier for indeterminate thyroid nodules: A clinical utility study in the Netherlands. Head Neck. 2023 Sep;45(9):2227-2236. doi: 10.1002/hed.27472. Epub 2023 Jul 25. PMID: 37490544.
15. Ahmadi S, Kotwal A, Bikas A, Xiang P, Goldner W, Patel A, et al. Outcomes of Cytologically Indeterminate Thyroid Nodules Managed With Genomic Sequencing Classifier. The Journal of Clinical Endocrinology & Metabolism. 2024; 109 (12): e2231–e2239. https://doi.org/10.1210/clinem/dgae112
16. Kim NE, Raghunathan RS, Hughes EG, Longstaff XR, Tseng CH, Li S, Cheung DS, Gofnung YA, Famini P, Wu JX, Yeh MW, Livhits MJ. Bethesda III and IV Thyroid Nodules Managed Nonoperatively After Molecular Testing With Afirma GSC or Thyroseq v3. J Clin Endocrinol Metab. 2023 Aug 18;108(9):e698-e703. doi: 10.1210/clinem/dgad181. PMID: 36995878; PMCID: PMC10438873.
17. Steward DL, Carty SE, Sippel RS, Yang SP, Sosa JA, Sipos JA, Figge JJ, Mandel S, Haugen BR, Burman KD, Baloch ZW, Lloyd RV, Seethala RR, Gooding WE, Chiosea SI, Gomes-Lima C, Ferris RL, Folek JM, Khawaja RA, Kundra P, Loh KS, Marshall CB, Mayson S, McCoy KL, Nga ME, Ngiam KY, Nikiforova MN, Poehls JL, Ringel MD, Yang H, Yip L, Nikiforov YE. Performance of a Multigene Genomic Classifier in Thyroid Nodules With Indeterminate Cytology: A Prospective Blinded Multicenter Study. JAMA Oncol. 2019 Feb 1;5(2):204-212. doi: 10.1001/jamaoncol.2018.4616.
18. Jug R, Foo WC, Jones C, Ahmadi S, Jiang XS. High-risk and intermediate-high-risk results from the ThyroSeq v2 and v3 thyroid genomic classifier are associated with neoplasia: Independent performance assessment at an academic institution. Cancer Cytopathol. 2020 Aug;128(8):563-569. doi: 10.1002/cncy.22283. Epub 2020 Apr 27. PMID: 32339438.
19. Carty SE, Ohori NP, Hilko DA, McCoy KL, French EK, Manroa P, Morariu E, Sridharan S, Seethala RR, Yip L. The Clinical Utility of Molecular Testing in the Management of Thyroid Follicular Neoplasms (Bethesda IV Nodules). Ann Surg. 2020 Oct;272(4):621-627. doi: 10.1097/SLA.0000000000004130. PMID: 32773640.
20. Chen T, Gilfix BM, Rivera J, Sadeghi N, Richardson K, Hier MP, Forest VI, Fishman D, Caglar D, Pusztaszeri M, Mitmaker EJ, Payne RJ. The Role of the ThyroSeq v3 Molecular Test in the Surgical Management of Thyroid Nodules in the Canadian Public Health Care Setting. Thyroid. 2020 Sep;30(9):1280-1287. doi: 10.1089/thy.2019.0539. Epub 2020 May 5. PMID: 32242511.

21. Desai D, Lepe M, Baloch ZW, Mandel SJ. ThyroSeq v3 for Bethesda III and IV: An institutional experience. Cancer Cytopathol. 2021 Feb;129(2):164-170. doi: 10.1002/cncy.22362. Epub 2020 Oct 8. PMID: 33030808.
22. Gajzer DC, Tjendra Y, Kerr DA, Algashaamy K, Zuo Y, Menendez SG, Jorda M, Garcia-Buitrago M, Gomez-Fernandez C, Velez Torres JM. Probability of malignancy as determined by ThyroSeq v3 genomic classifier varies according to the subtype of atypia. Cancer Cytopathol. 2022 Nov;130(11):881-890. doi: 10.1002/cncy.22617. Epub 2022 Jul PMID: 35775861.
23. Tjendra Y, Kerr DA, Zuo Y, Menendez SG, Jorda M, Gomez-Fernandez C, Velez Torres JM. Probability of malignancy and molecular alterations as determined by ThyroSeq v3 genomic classifier in Bethesda Category IV. Cancer Cytopathol. 2023 Sep;131(9):586-595. doi: 10.1002/cncy.22737. Epub 2023 Jun 26. PMID: 37358081.
24. Sirotnikov S, Griffith CC, Lubin D, Zhang C, Saba NF, Li D, Kornfield A, Chen A, Shi Q. ThyroSeq overview on indeterminate thyroid nodules: An institutional experience. Diagn Cytopathol. 2024 Jul;52(7):353-361. doi: 10.1002/dc.25311. Epub 2024 Mar 30. PMID: 38554032.
25. Yang SP, Nga ME, Bundele MM, Chiosea SI, Tan SH, Lum JHY, Parameswaran R, Lim MY, Li H, Cheah WK, Sek KS, Tan ATH, Loh TKS, Ngiam KY, Tan WB, Huang X, Ho TWT, Lim KH, Lim CM, Singaporewalla RM, Rao AD, Rao NCL, Chua DYK, Chin DC, Wald AI, LiVolsi VA,
26. Lévesque F, Payne R, Beaudoin D, Boucher A, Fortier PH, Massicotte MH, et al. Publicly Funded Molecular Testing of Indeterminate Thyroid Nodules: Canada's Experience, The Journal of Clinical Endocrinology & Metabolism. 2025 Apr; 110 (4): e1031–e1037, https://doi.org/10.1210/clinem/dgae355
